# Clay-Based Technologies for Produced Water Treatment: A Bibliometric Analysis of Global Trends and SDG Contributions (1996–2025)

**DOI:** 10.1021/acsomega.5c10571

**Published:** 2026-06-11

**Authors:** Somaya Ahmed, Saeed Al-Meer, Ahmed Ben Ali, Mustafa S. Nasser, Ibnelwaleed A. Hussein

**Affiliations:** † Gas Processing Center, 61780 Qatar University, P.O. Box 2713 Doha, Qatar; ‡ Department of Chemistry and Earth Sciences, Qatar University, P.O. Box 2713 Doha, Qatar; § Department of Chemical Engineering, Qatar University, P.O. Box 2713 Doha, Qatar

## Abstract

The oil and gas sector has experienced substantial growth in recent decades, leading to a significant increase in produced water containing hazardous pollutants that frequently surpass environmental standards and regulations. This study underscores the importance of developing sustainable and cost-effective treatment methods, particularly clay-based approaches that are renowned for their availability, affordability, and environmental sustainability. A bibliometric analysis was conducted to examine global trends in clay-based wastewater treatment from 1996 to 2025, using data from Web of Science and Scopus, which identified 1022 relevant publications. Visualization tools such as VOSviewer and SciVal were employed to map research networks and identify leading authors, institutions, countries, and key themes. The results demonstrate a considerable rise in scholarly articles on the use of clay-based materials for treating produced water, with China emerging as the foremost contributor. Keyword analysis revealed primary themes including adsorption, clay-based composites, and wastewater reuse, indicating a transition toward more sustainable water management practices. This study further emphasizes the vital role of clay-based technologies in achieving Sustainable Development Goal 6 by fostering water reuse and pollution reduction. It also highlights the necessity for continued research on produced water to diminish contamination levels and operational costs, offering valuable insights for researchers, policymakers, and industry stakeholders regarding material innovation, hybrid treatment systems, and techno-economic assessments to enhance scalability and efficiency.

## Graphical abstract

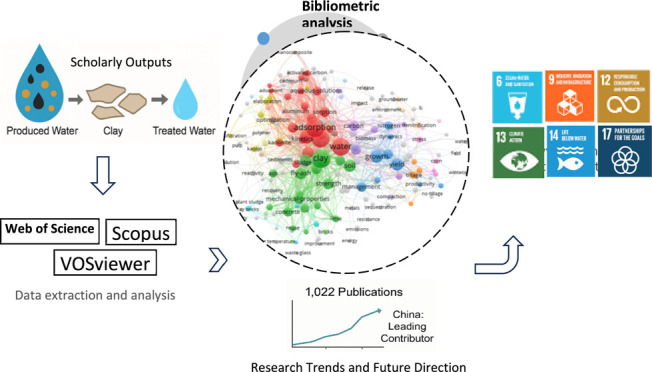

## 1 Introduction

Ensuring access to clean, high-quality water has become one of the most urgent global challenges. The rising demand for energy is driving oil and gas production, which, in turn, increases the volume of produced water (PW). This wastewater often contains high levels of hydrocarbons, salinity, and heavy metals, posing significant environmental challenges.^1^ Effective treatment of PW is crucial for reducing environmental impacts, promoting water reuse, and facilitating resource recovery, all of which are essential in addressing global water scarcity and supporting sustainable development.^2^ Conventional treatment methods, including biological processes, chemical and advanced oxidation, adsorption, and coagulation, exhibit well-documented limitations when applied to complex PW streams.^3^ High salinity can inhibit biological pathways; oxidation processes may yield hazardous byproducts, and many traditional operations are inefficient, costly, or energy-intensive for removing heavy metals, emulsified oil, and certain dissolved organics.^3^ Among the various treatment technologies, clay-based materials have emerged as promising, cost-effective, and environmentally friendly adsorbents due to their abundance, low toxicity, and high efficiency in removing a wide range of pollutants from PW.^4^

In recent years, there has been an increasing focus on aligning PW management practices with the United Nations’ Sustainable Development Goals (SDGs), especially SDG 6, which strives to ensure the availability and sustainable management of water and sanitation for everyone.^5^ The adoption of advanced treatment methods, such as those using clay and its composites, helps to reduce pollution, promote water reuse, and support broader environmental and social objectives outlined in the SDGs. Clay-based materials, including clay–polymer nanocomposites and geopolymers, offer notable advantages, such as global availability, high adsorption capacity, and low cost. Studies have shown that these materials effectively remove both organic and inorganic contaminants from water and wastewater. Their application also promotes recycling, supports sustainable water management, and mitigates water scarcity, which are key objectives of SDG 6.^6,7^ A comprehensive understanding of research trends, key contributors, and emerging themes in clay-based PW treatment remains limited. However, bibliometric analysis offers a systematic methodology for quantitatively mapping the evolution, impact, and collaborative networks in the scientific literature of this field.^8^ Bibliometric analysis is a quantitative method for analyzing data from various areas of study, evaluating aspects such as publications and citation patterns. This type of study began in the 1950s.^9^ It enables the assessment of document types, collaborations, publication counts, keywords, and countries that make significant contributions to a research topic. Applied to indexed literature on adsorption-based methods using clay and its composites, bibliometrics can reveal leading contributors, impactful venues, emergent hotspots, and methodological gaps that shape water treatment research.^10^

This paper reviews the research trends and key features of using clay to treat PW. Using bibliometric analysis, it examines the growth of scientific work in this area, highlights the challenges, and identifies new opportunities. The aim is to help researchers and stakeholders understand the current knowledge, find gaps, and suggest areas for future study. The paper also focuses on how these clay-based treatments support the SDGs, particularly those related to sustainable water management, to inform policy and future developments.

## 2 Background

### 2.1 Characteristics of PW

#### 2.1.1 Chemical and Physical Composition of PW

PW is a significant byproduct of oil and gas operations, with its composition influenced by several factors such as the age of the well, its location, depth, reservoir geology, and extraction techniques used.^11^ Unlike ordinary seawater, which has an average salinity of about 35,000 ppm, PW often exhibits salinity levels ranging from 23,000 to 67,300 ppm and, in extreme cases, can reach up to 195 ppt, making it exceptionally briny.^2^ This elevated salinity results from high concentrations of dissolved solids, particularly sodium (up to 141,000 ppm, compared to ∼10,565 ppm in seawater) and chloride (up to 25,800 ppm, compared to ∼18,982 ppm in seawater). PW also contains elevated levels of other ions such as calcium, magnesium, bromide, and potassium.^12^ Beyond naturally occurring salts, PW carries a complex mixture of contaminants introduced during extraction and processing, including hydrocarbons, heavy metals, polycyclic aromatic hydrocarbons (PAHs), and chemical additives. The exact composition varies widely, depending on geological formations, operational chemicals, and separation methods employed. Discharging this highly saline and contaminated water without adequate treatment poses severe environmental risks, particularly to marine ecosystems, where toxic substances can bioaccumulate in organisms over time.^12,13^ Consequently, the effective management of this heterogeneous wastewater stream is critical for environmental protection and sustainable resource use.

#### 2.1.2 Differences between PW from Oil and Gas Fields

Although both oil and gas fields produce PW with similar contaminants, their levels vary greatly. PW from gas fields generally shows higher concentrations of total organic carbon (TOC), total suspended solids (TSS), total dissolved solids (TDS), and chemical oxygen demand (COD) compared with PW from oil fields. Sometimes, gas field PW also contains elevated levels of hydrocarbons and dissolved acidic gases, which makes treatment more difficult. However, PW from oil fields often exceeds environmental safety limits, requiring proper treatment before reuse or disposal.^7^

### 2.2 PW Treatment Technologies

Efficient treatment technologies are essential for managing the large volumes of PW generated by oil and gas operations, given the complex and variable compositions of these effluents. Various physical methods, such as flotation, filtration, and gravity separation, are commonly used to remove oil and suspended solids.^14^ Chemical treatments like oxidation, coagulation, and pH adjustment facilitate the removal of organic substances, microbiological pollutants, and fine particles.^15^ Additionally, membrane-based techniques, including reverse osmosis, nanofiltration, and ultrafiltration, are effective for desalination and organic contaminant removal.^16,17^ Biological processes, such as membrane bioreactors, activated sludge systems, and artificial wetlands, utilize microbial activity to degrade contaminants.^18,19^ Thermal processes like evaporation, distillation, and zero liquid discharge (ZLD) focus on water recovery and waste concentration.^20–22^ Adsorption treatment using materials such as zeolites, clays, chitosan, activated carbon, polymers, resins, activated alumina, silica gel, molecular sieves, organoclays, and ion exchange resins targets specific pollutants like organics and salts.^23,24^ Figure 1 provides an overview of the various technologies used to treat PW.

**Figure 1:**
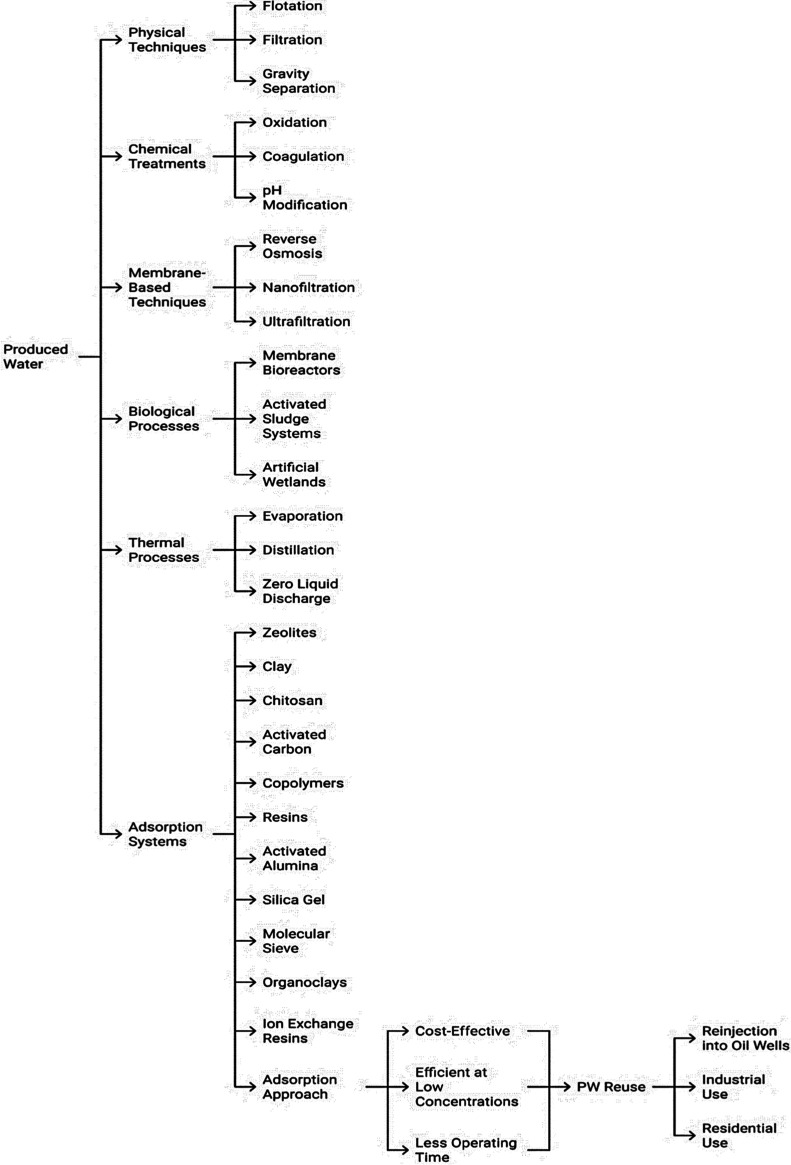
PW treatment technologies.

The adsorption approach is widely considered one of the most practical and economical methods for treating PW.^25^ Compared to other techniques, adsorption is generally more economical, performs effectively at low pollutant concentrations, and requires shorter operating times.^25–27^ These advantages reduce overall treatment costs, which in turn enhance the feasibility of PW reuse in applications such as reinjection into oil reservoirs or use in industrial and even residential settings.^28^ Such reuse strategies not only support sustainable resource management but also create economic value, transforming PW from a waste stream into a valuable resource and incentivizing industries to invest in treatment technologies.

Recent research shows that graphene-based adsorbents function very effectively. According to Thi Mai et al.,^28^ functionalized graphene (FG) nanosheets demonstrated exceptional adsorption capacities, including removal efficiencies of 95.56% for Pb^2+^, 95.82% for methyl orange, 99.95% for enrofloxacin, and 98.93% for methylene blue. The work demonstrated high performance and sustainability by using graphite waste from spent household batteries in a one-step, nonelectrochemical synthesis. Moreover, Azmoon et al.^29^ demonstrated the potential of MOF-based composites for complicated wastewater matrices by developing the novel ternary heterostructure MIL-101­(Cr)/Fe_3_O_4_/g-C_3_N_4_ for oilfield PW treatment.

Although advanced synthetic adsorbents offer higher removal efficiency, their costly synthesis process and limited scalability prevent widespread industrial use. Consequently, naturally occurring clay minerals provide a cost-effective and easily accessible alternative for large-scale PW treatment.

### 2.3 Clay-Based Treatment Methods

Clays and clay minerals have been widely used as adsorbents in water treatment due to their abundance, affordability, and environmentally friendly characteristics.^25^ Their layered structure, high surface area, and ion-exchange capacity make them very suitable for removing a wide range of contaminants. While natural and modified clays, such as those treated by heating, surfactants, acid activation, or functionalization, have shown significant potential, they sometimes face limitations in reusability and efficiency for certain organic pollutants.^30^ Recent advancements have focused on developing clay-based composite materials that overcome the limitations of traditional clays. These composites combine the natural advantages of clay with enhanced structural and functional properties, resulting in significantly higher adsorption capacity, improved recovery from water systems, and superior physical and chemical stability.^31^

Clay minerals such as bentonite, montmorillonite, and kaolinite are acknowledged as affordable, readily available, and highly efficient adsorbents for eliminating both organic and inorganic contaminants from water and wastewater.^32^ The charged and layered platelets comprising the chemical structure of clay minerals impart them with unique swelling and ionic exchange properties that promote the adsorption of various inorganic and organic contaminants in wastewater.^33^ The sorption capabilities of clays can be enhanced through various methods, including intercalation, acid treatment, calcination, and pillaring.^34^

Although clay minerals and clay composites are commonly used in the literature to remove heavy metals, pharmaceutical contaminants, and dyes from water, their recent applications in separating oil from water and in wastewater treatment have been limited.^35^ For instance, Akpomie et al.^36^ applied natural clay from Nigeria to clean an oil spill. The clay efficiently removed crude oil, exhibiting a sorption capacity of 6.9 g/g at 27 °C and pH 4. The clay was also regenerable and could be used as an adsorbent up to 3 times without diminishing its sorption capacity. Similarly, Bentonite/metal oxide complexes have been reported in several studies to exhibit higher oil adsorption capacities than the individual constituents.^37^ For example, bentonite/iron oxide composites studied by Ewis et al.^37^ demonstrated a diesel oil removal efficiency of 71% from oil-in-water emulsion. This enhanced sorption capacity is attributed to the greater presence of functional groups on the composite surface compared to the clay alone, as well as increased surface hydrophobicity.^32^

Despite these advantages, clay-based adsorption presents notable technical limitations that warrant critical consideration. First, clay performance is highly sensitive to ionic strength: at TDS levels exceeding 50,000 ppm, characteristic of deep-formation PW, competitive ion effects from Na^+^, Ca^2+^, and Mg^2+^ substantially reduce adsorption capacity, as the background electrolyte competes with target contaminants for active surface sites on clay minerals.^38,39^ This constraint is not encountered with membrane-based systems such as reverse osmosis, which maintain >95% desalination efficiency independent of ionic composition.^16,17^ Second, natural clays generally exhibit limited reusability; for instance, magnetic montmorillonite retains a regeneration rate above 80% after three calcination cycles, beyond which structural degradation leads to measurable capacity loss,^30^ and organobentonite desorption efficiency declines from 99.7% to 89.3% after four regeneration cycles.^40,41^ Third, the capacity of unmodified clay to adsorb emerging contaminants such as PFAS, microplastics, and pharmaceutical residues remains poorly characterized; unmodified montmorillonite exhibits a PFOS binding capacity of only 0.20 mol/kg compared to 0.45 mol/kg for caffeine-amended clay and a GenX binding capacity of 0.15 mol/kg versus 1.17 mol/kg for the modified counterpart,^28^ indicating that surface modification is essential for emerging-contaminant removal. Finally, while clay-based systems offer clear cost advantages, organoclay removal efficiency has been reported to be 7 times higher than that of activated carbon at a lower cost, and the estimated global treatment cost of PW reaches USD 40 billion per annum.^42^ The absence of standardized techno-economic assessments and life-cycle analyses in the current literature makes it difficult to substantiate robust cost comparisons with competing technologies.^43^ Addressing these limitations through material engineering, such as nanomodification, polymer functionalization, and hybrid system design, represents a priority direction for future research. Table 1 summarizes PW treatment techniques.

**Table 1:** Comparative Assessment of Clay-Based and Alternative PW Treatment Technologies

technology	removal efficiency	salinity tolerance	energy requirement	relative cost	scalability	key limitation	refs
clay adsorption (natural)	up to 90% heavy metals; 71% oil	moderate suppressed at TDS > 50,000 ppm	low	very low	moderate	limited reusability (∼3 cycles); salinity sensitivity	^35,37^
clay composites (modified)	up to 90%	improved vs natural clay	low–moderate	low–moderate	moderate	synthesis complexity; spent clay disposal	^40^
activated carbon	75–96% heavy metals; 85–98% organics	high	moderate (regeneration energy required)	moderate–high	high	high production and regeneration cost	^44^
graphene-based adsorbents	95–99.95%	high	low	very high	low fragility limits scale-up beyond lab	cost-prohibitive at scale; fragility	^45^
reverse osmosis/NF	up to 99% salt rejection; >95% desalination	very high	very high	high	high	membrane fouling; brine disposal (∼33% of OPEX)	^46^
biological treatment	average COD removal 73% (TDS < 50,000 ppm); drops to 54% (TDS > 50,000 ppm)	low-inhibited at TDS > 50,000 mg L^–1^	moderate	moderate	high	microbial inhibition by salinity; fouling; acclimation time	^47^
thermal/ZLD	up to 98.6% water recovery	very high	very high (7.3–65 kWh m^–3^)	very high ($0.8–10 m^–3^ MVC)	high	energy-intensive; high CAPEX	^48^
coagulation/flocculation	70–99% TSS; 46–88% COD	moderate	low	low	high	sludge disposal; chemical addition required	^49^

## 3 Methodology

### 3.1 Data Collection

The data used in this study were obtained from two internationally recognized academic databases, Web of Science (WoS) and Scopus, which are known for their vast collections of scholarly literature. Only publications that particularly discussed the use of clay-based techniques to treat PW were selected for investigation. VOSviewer was used to visualize and create bibliometric maps using information taken from bibliographic files obtained from WoS and Scopus. Figure 2 illustrates the general approach.

**Figure 2:**
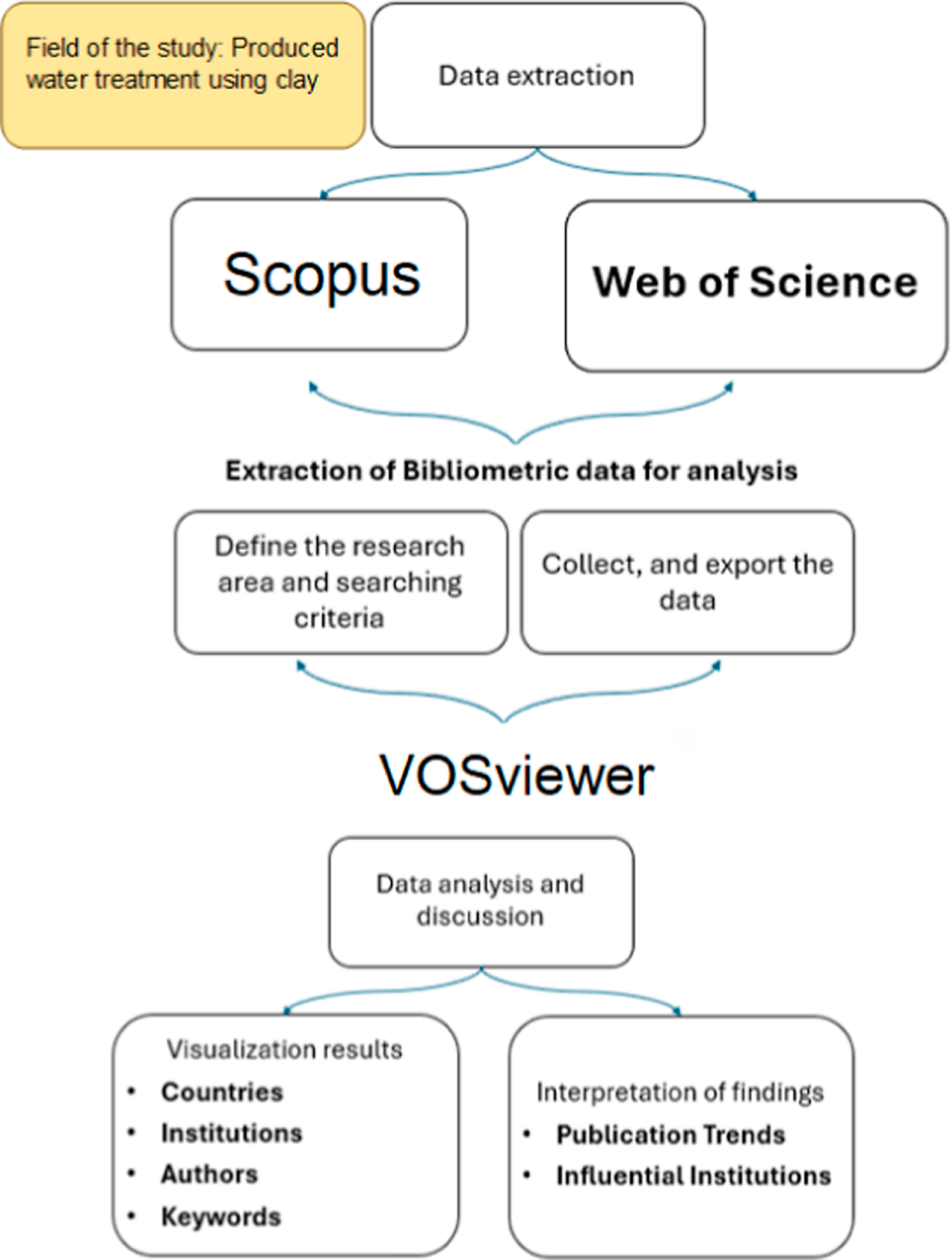
Methodology of the review.

### 3.2 Search Strategy

To collect data for this study, related articles were searched from Scopus and WoS and accessed on April 20, 2025. The complete Boolean search strings used in Scopus were as follows: TITLE-ABS-KEY ((“produced water” OR “oilfield wastewater”) AND (“clay” OR “bentonite” OR “natural clay”) AND (“treatment” OR “adsorption” OR “removal”)). The equivalent search query applied in Web of Science (WoS) was: ALL = (“produced water” OR “oilfield wastewater″) AND (“clay” OR “natural clay”) AND (“treatment” OR “adsorption” OR “removal”)). In both databases, the search was restricted to English-language documents and limited to the publication date range 1996–2025. 1996 was chosen as the lower boundary because it is the earliest year allowed by SciVal while capturing the first relevant publications. This period also coincides with the growing global regulatory pressure on produced-water discharge management in the oil and gas sector, which has stimulated systematic academic interest in low-cost, naturally abundant treatment alternatives, such as clay minerals. To validate the comprehensiveness and consistency of the retrieval strategy, both databases were queried independently using identical search criteria, and the results were cross-checked.

### 3.3 Limitations of the Bibliometric Approach

The bibliometric approach, while systematic, possesses several inherent limitations that may influence the outcomes. Relying exclusively on the Scopus and WoS databases excludes scholarly works not indexed in these platforms, including regional journals and patents, potentially leading to an under-representation of contributions from specific areas. Furthermore, focusing solely on English-language publications introduces substantial language bias, potentially overlooking valuable research from non-Anglophone nations. The specificity of the search query also increases the likelihood of overlooking pertinent literature that employs different terminology for key concepts. Finally, the analysis is predominantly quantitative, emphasizing metrics that may not adequately capture the qualitative impact, originality, or practical significance of the research or account for the contextual factors behind citation patterns.

## 4 Results and Discussion

### 4.1 Publication Trends

The number of research articles on the treatment of industrial wastewater, including PW, has been steadily increasing. Of particular interest are studies on adsorption technologies, including those using clay-based materials. Figure 3 displays the annual number of publications retrieved from Scopus between 1996 and 2025 (to match SciVal limitations). A total of 1022 publications on PW treatment using clay were retrieved, averaging approximately 34 publications per year, with a consistent rise observed over time. The highest annual output was recorded in 2023, with 298 scholarly outputs. This growth is indicative of a deliberate and accelerating shift in the scientific community’s focus toward sustainable, cost-effective water treatment solutions, beyond a general expansion of academic output. The post-2016 acceleration aligns with the adoption of the SDGs (especially SDG 6 on Clean Water and Sanitation) and the Paris Agreement, which collectively redirected international research funding toward environmentally responsible water technologies. Concurrently, increasingly stringent produced-water discharge regulations, including the EU Water Framework Directive and the US EPA effluent guidelines for oil and gas extraction, have intensified the demand for cost-effective treatment alternatives, further motivating research into clay-based adsorption. These converging regulatory, policy, and environmental pressures collectively underlie the observed growth trajectory as PW volumes continue to rise in parallel with expanding global oil and gas activity.

**Figure 3:**
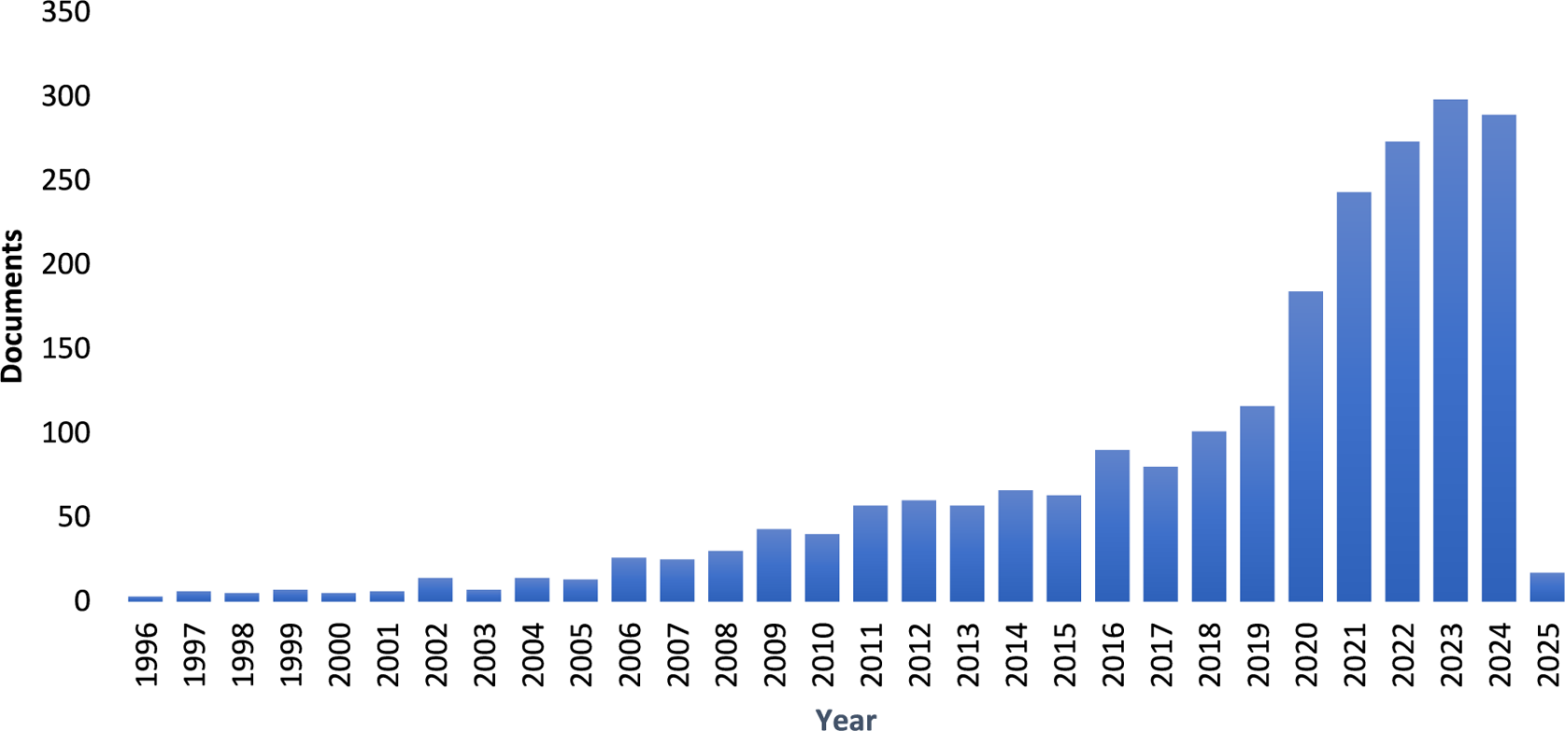
Annual scholarly outputs of PW treatment using clay.

A total of 1022 papers on PW treatment using clay were retrieved from Scopus, and 1003 from WoS, confirming the reliability of both databases. About 80.3% of these are research articles and 1.8% are review papers. The remaining 17.9% include conference papers, editorials, errata, letters, notes, and conference reviews, as shown in Figure 4. Several experiment-driven fields continue to examine clay-based PW treatment, as made evident by numerous studies.

**Figure 4:**
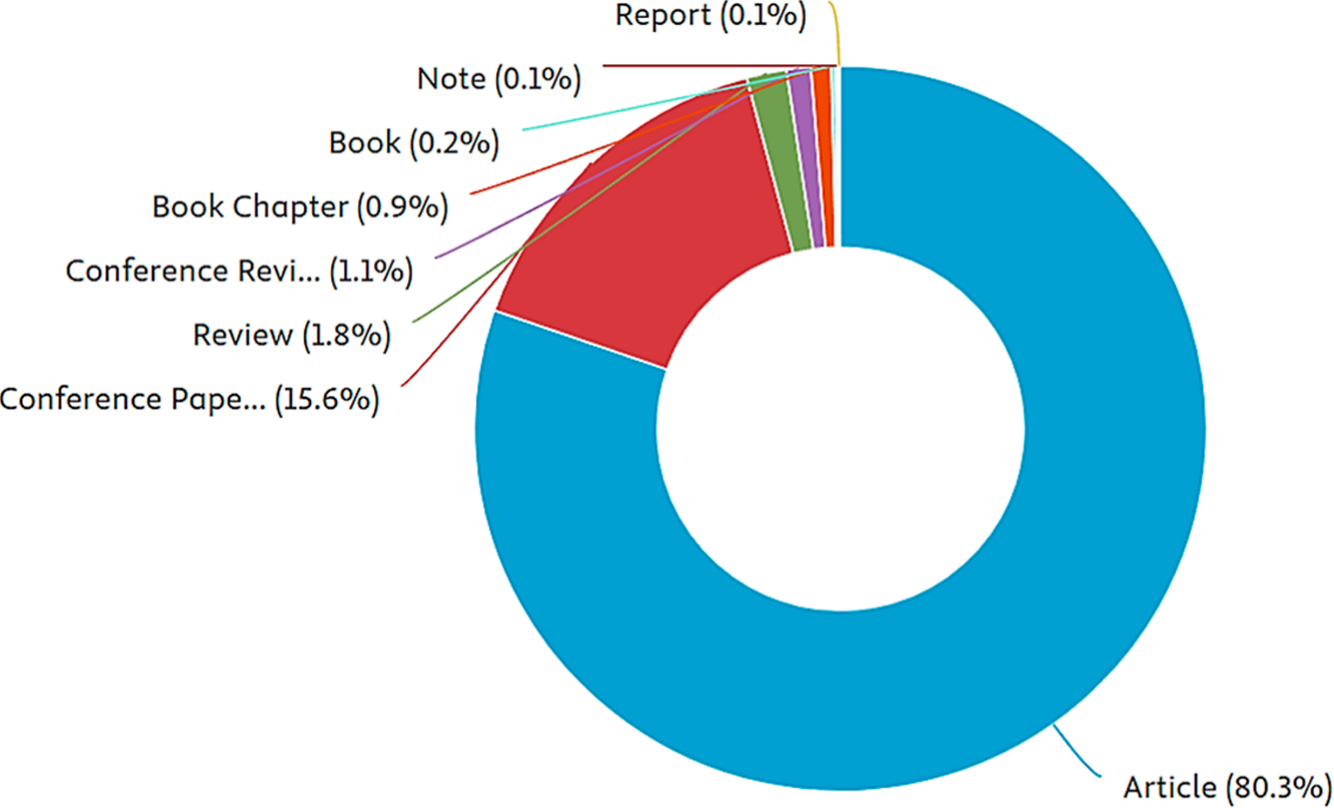
Publication types.

### 4.2 Influential Authors and Institutions

The collaboration network illustrated in Figure 5 highlights the institutional framework of clay-based PW treatment research, where node size indicates publication output and connecting lines show coauthorship partnerships. China leads in publication volume (∼12.7% of the total outputs), driven by ongoing national investment through programs such as the National Natural Science Foundation of China (NSFC) and targeted water action plans, indicating a strategic focus on low-cost, scalable treatment technologies in response to domestic water scarcity and expanding oil and gas production. Although the United States and Europe generally produce publications with higher citation impact, the increasing density of international collaborative ties signals a more globalized research effort with implications for technology adoption across various regulatory environments.

**Figure 5:**
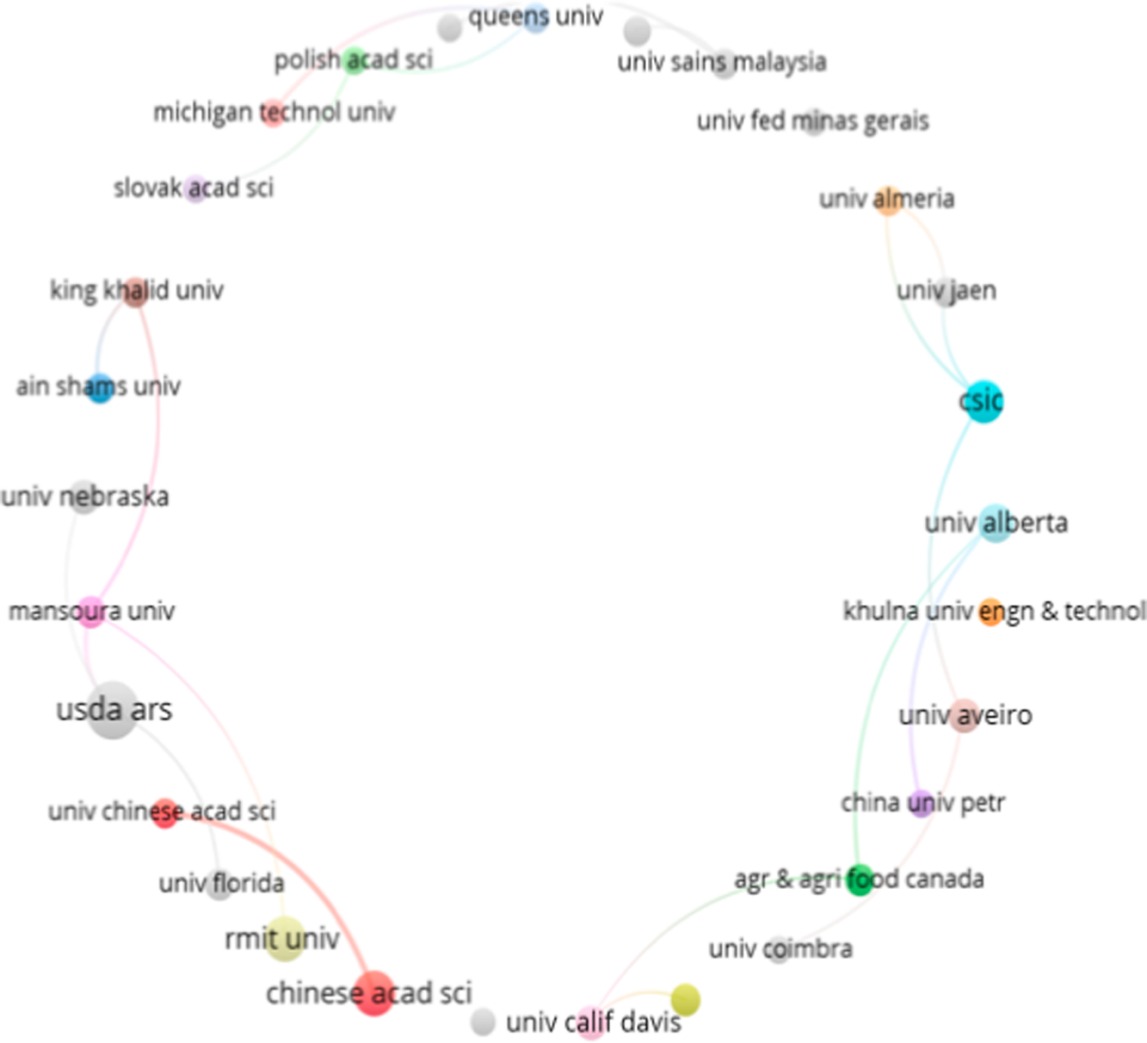
Influential institutions.

At the institutional level, the Chinese Academy of Sciences is the top contributor with 19 papers, reflecting its government-funded mandate for environmental research across dedicated water treatment and clay mineralogy programmes. The Spanish National Research Council (CSIC) (13 papers) acts as a key bridging node linking European clusters to global networks, consistent with its long-standing expertise in clay surface chemistry. The USDA Agricultural Research Service (12 papers) and the University of Alberta (8 papers) exemplify distinctly North American drivers: assessment of agricultural PW reuse and water management in oil sands, respectively. Collectively, this institutional landscape reveals a research domain shaped as much by national resource pressures and regulatory requirements as by pure academic interest, a dynamic that directly influences how findings are prioritized and translated into industrial applications and practice.

The author’s collaboration network in Figure 6 reveals the key contributors and the collaborative dynamics shaping research on clay-based PW treatment. Abbas Mohajerani (4 publications) holds the most central position in the network, a status that extends beyond publication counts: his research connects environmental remediation and construction materials science, specifically the valorization of clay and treated sludge into bricks and concrete. This makes him a rare interdisciplinary connector between two communities that otherwise operate in parallel. Aruna Ukwatta (3 publications), affiliated with the same RMIT University group, reinforces this dual focus through extensive collaborative partnerships. The Spanish cluster, comprising Pilar Aranda, Sofia Payel, and Margarita Darder, reflects CSIC’s established expertise in clay surface chemistry and organoclay modification, consistent with the institutional prominence seen in Figure 5. The closely linked group of Nicky Eshtiaghi and Sujeeva Setunge similarly anchors RMIT’s contribution at the intersection of sustainable waste management and materials engineering. Notably, the relatively low individual publication counts across all prominent authors indicate that this field has not yet coalesced around a small number of dominant researchers but remains broadly distributed, a pattern that, while reflecting healthy diversity, also indicates limited cross-cluster collaboration and a fragmented knowledge base that could delay the translation of experimental findings into unified engineering frameworks.

**Figure 6:**
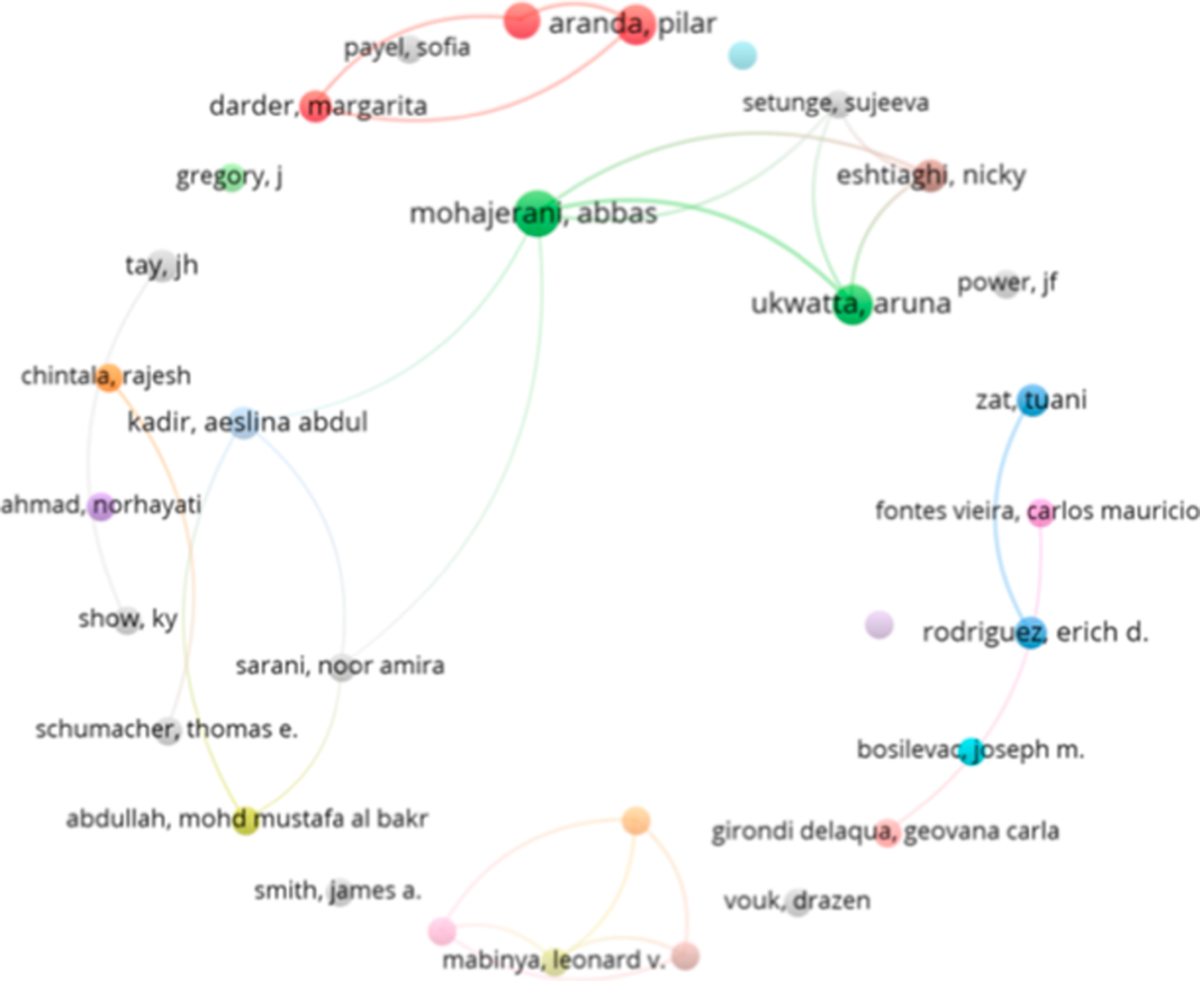
Influential authors.

### 4.3 Top Journals on Clay and Water Treatment

The journal distribution in Figure 7 highlights not only where this research is published but also who is reading it. Applied clay science leads with 21%, confirming the field’s identity within materials science rather than environmental engineering, which limits its reach to practitioners relying on outlets such as Water Research or Environmental Science & Technology that are absent from this landscape. The two agricultural journals, Agricultural Water Management and Soil and Tillage Research, each contribute 11%, collectively matching Applied Clay Science and confirming that PW reuse for irrigation is a coequal research driver, not a peripheral concern. Journal of Hazardous Materials (10%) and Chemosphere (8%) reflect a pollutant-risk framing, while Waste Management (8%) reinforces the circular economy dimension. The disproportionately small share of Science of the Total Environment (4%) suggests that holistic, systems-level assessments remain underrepresented. Overall, this distribution points to a productive but discipline-silenced community whose findings are not yet reaching the engineering and policy audiences best positioned to implement them.

### 4.4 Key Themes and Topics

The keyword co-occurrence network illustrates the thematic landscape and connections within research on clay-based PW treatment, as shown in Figure 8. Key terms such as “adsorption”, “water”, “clay,” “sludge,” and “Soil” stand out as the main research focus, with their importance indicated by node size. The network is divided into three distinct thematic groups, each representing a specific research area.

**Figure 7:**
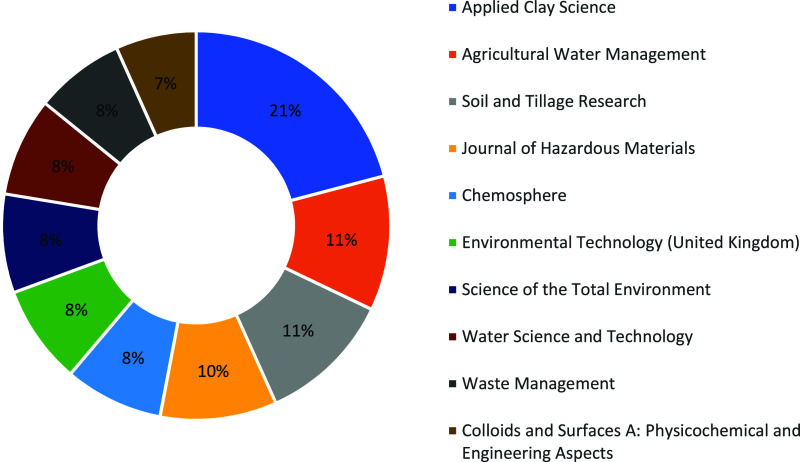
Top journals on clay and water treatment.

**Figure 8:**
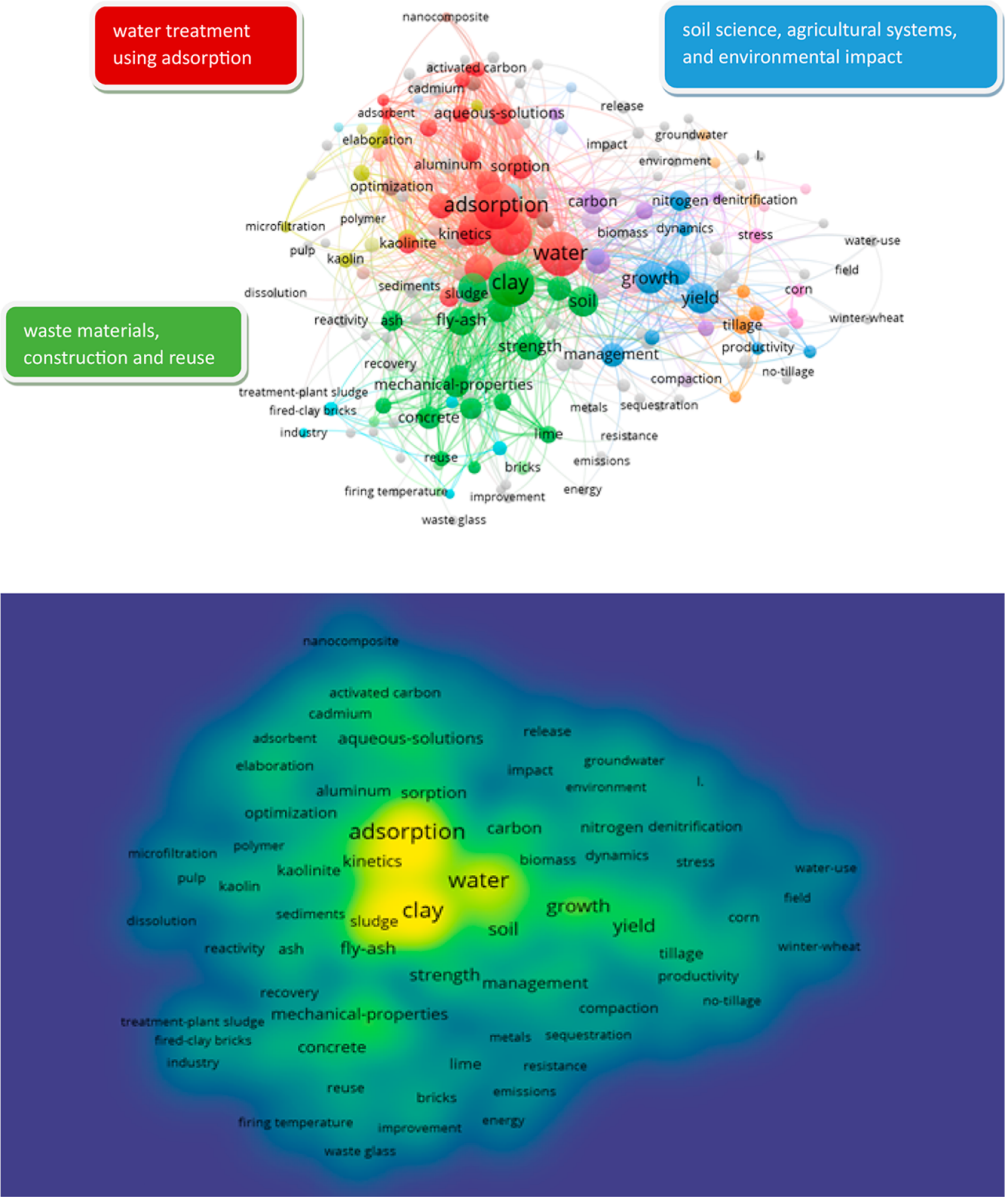
Top keywords associated with PW treatment using clay.

The red cluster, which groups adsorption mechanisms with sorption studies, represents the most mature and well-developed strand of the literature. The green cluster, linking clay materials with construction applications and waste valorization (clay bricks, concrete, and reuse), signals an emerging research paradigm aligned with circular economy principles, transforming spent or modified clay adsorbents into value-added construction products rather than treating them as secondary waste. The blue cluster, encompassing soil science and agricultural systems, reflects growing recognition that PW treatment and reuse must be evaluated within broader land and water management frameworks, particularly in arid regions, where PW represents a viable irrigation source. Critically, the co-occurrence network also reveals a notable gap: keywords associated with techno-economic analysis, life cycle assessment, and scale-up are conspicuously absent from the high-frequency nodes, indicating that despite extensive laboratory-level investigation, the transition toward economically validated, large-scale clay-based treatment systems has received disproportionately little attention in the analyzed corpus.

The SciVal word cloud in Figure 9 confirms and extends the thematic patterns identified in the VOSviewer network but offers an additional analytical dimension: the keyword frequency distribution reveals not just what the field studies, but also how its material focus has evolved. The dominance of broad terms like “Clay,” “Adsorption,” “Water Treatment,” and “Wastewater Treatment” establishes the core identity of the field, while the copresence of natural clay minerals (kaolinite and bentonite) alongside engineered carbonaceous materials (activated carbon and biochar) indicates a research shift from studying raw natural clays to hybrid and composite adsorbent systems with improved performance characteristics. The prominence of “Heavy Metal” and “Organic Pollutant” as contaminant descriptors, which appear more frequently than “Produced Water,” specifically suggests that PW treatment remains a subniche within the broader clay-based wastewater treatment literature, pointing to an opportunity to develop more PW-specific research frameworks rather than extrapolating from general wastewater studies. The appearance of construction terms (“Clay Brick,” “Concrete,” and “Reuse”) alongside remediation vocabulary reinforces the circular economy dimension identified in Figure 8, while agricultural terms (“Soil”, “Irrigation”, and “Water Use Efficiency”) reflect the field’s expanding scope toward integrated water-soil-food nexus management. Together, the word cloud and VOSviewer map support a research landscape that is broadening in material complexity and application context but remains anchored to laboratory-scale adsorption studies, highlighting the persistent gap between experimental knowledge generation and practical system design.

**Figure 9:**
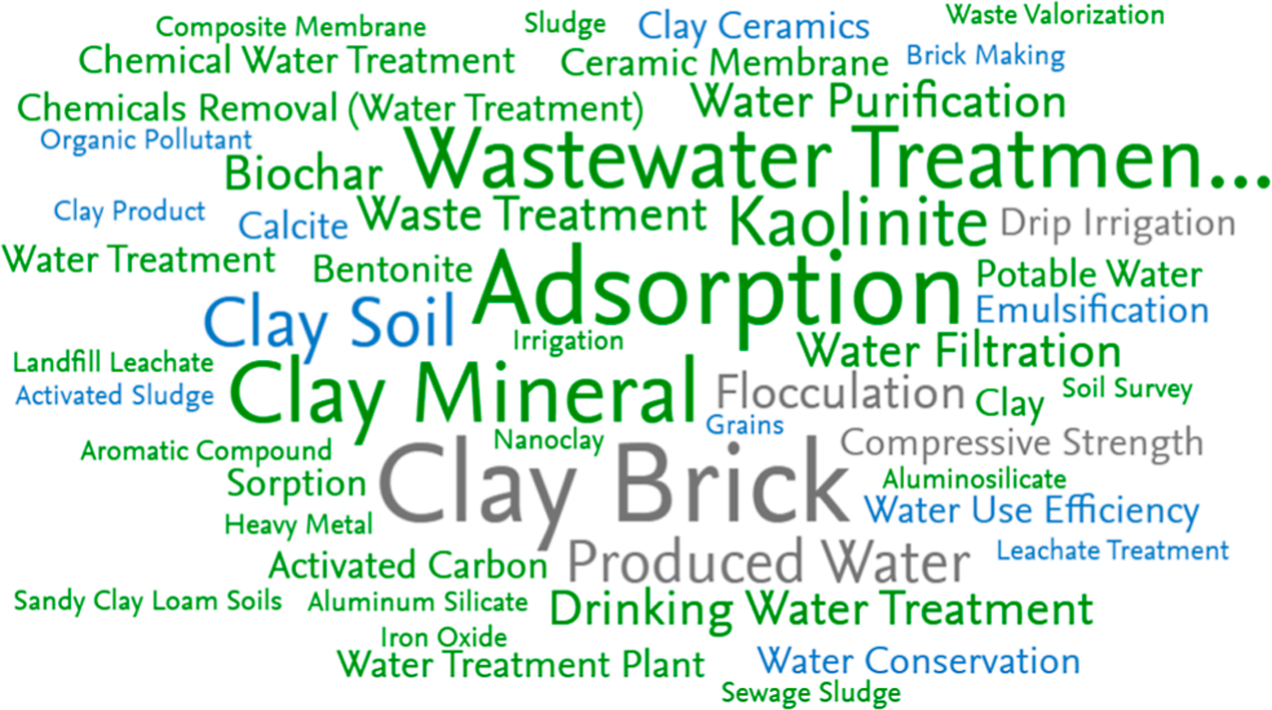
Top keywords extracted from SciVal.

The SciVal subject area wheel in Figure 10 represents the disciplinary distribution of all publications, confirming the inherently interdisciplinary nature of clay-based PW treatment research across materials science, environmental engineering, and applied chemistry. The most analytically notable finding is the prominence of the “Compressive Strength, Sintering, and Thermal Conductivity” cluster, which ranks at the 97.38th percentile globally, placing it among the top 3% of all research clusters worldwide in terms of visibility and citation impact. Despite comprising only 37 publications (2.70%), this cluster’s exceptional prominence shows that the most internationally visible work in this field focuses on materials engineering rather than environmental remediation. This is analytically significant: it indicates that clay-based research achieves its highest global impact not solely through water treatment performance studies but through investigations of the postuse structural properties of clay, specifically how spent or modified clay adsorbents can be reprocessed via sintering into construction materials (bricks, ceramics) with defined mechanical and thermal performance benchmarks. This finding directly supports the circular economy dimension identified in Figures 7 and 8 and has important implications for research strategy: increasing efforts toward the materials engineering pathway, which characterizes spent clay adsorbents as construction feedstocks, may yield disproportionately high international visibility and citation impact compared to incremental adsorption performance studies.

**Figure 10:**
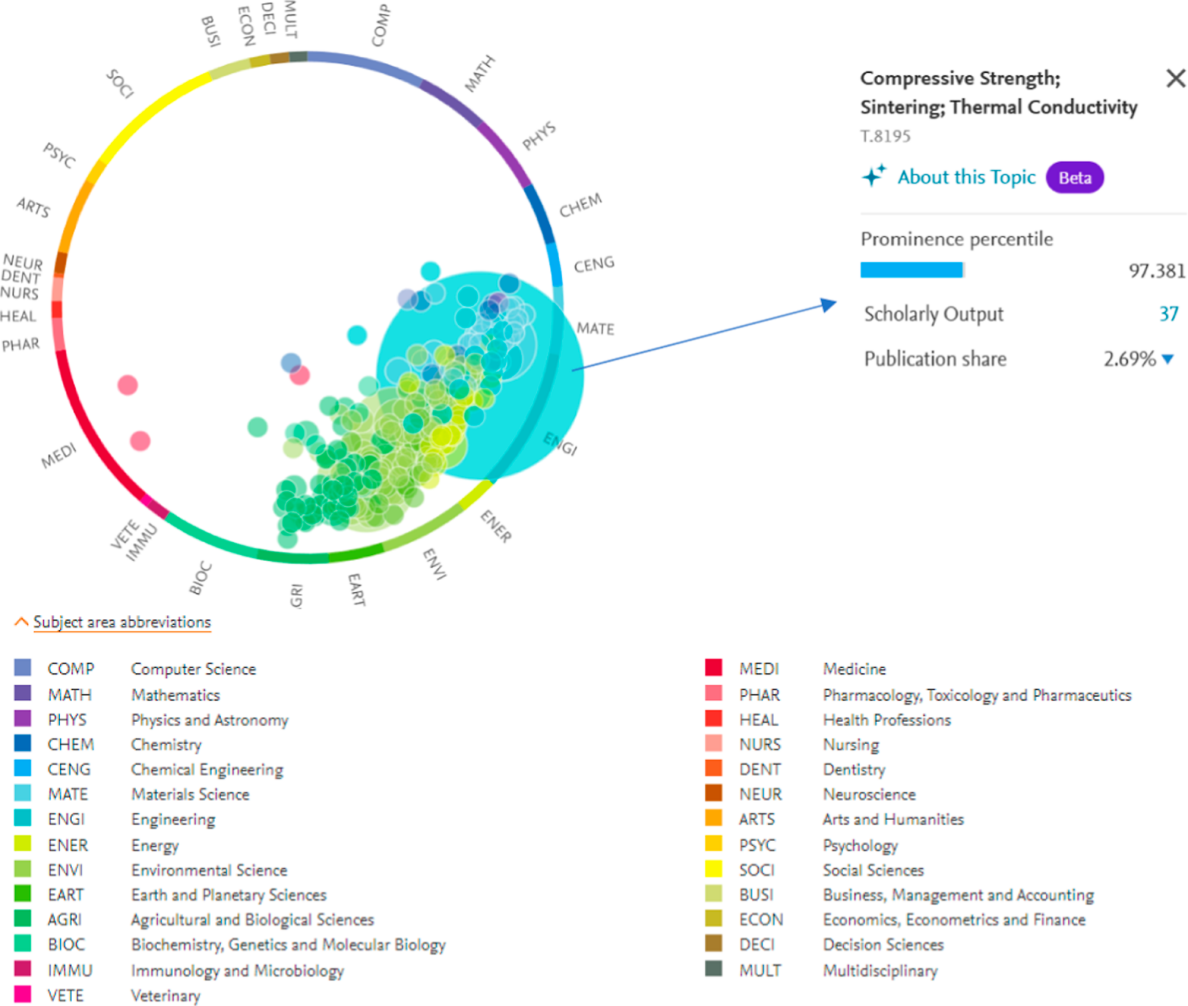
Topic clusters extracted from SciVal.

## 5 Adsorption Mechanisms: Bibliometric-Anchored Technical Analysis

The keyword co-occurrence network in Figure 8 identifies adsorption mechanisms as the dominant thematic cluster across the 1022 papers. This reflects a global pattern also reported in bibliometric analyses of seaweed biosorption and tetracycline adsorption, where adsorption modeling consistently forms the methodological core of the field.^50^ The following subsections integrate the theoretical basis of each model with its observed bibliometric frequency and temporal evolution within the clay-based PW treatment literature.

### 5.1 Isotherms

Isotherm models describe the equilibrium relationship between the adsorbate concentration in solution and the quantity retained on the solid surface at constant temperature, providing insight into surface heterogeneity, adsorption capacity, and interaction strength. These parameters dominate the red keyword cluster centered on adsorption, clay, surface area, and composites in the VOSviewer map (Figure 8), confirming isotherm analysis as the principal methodological focus of the scholarly outputs.

#### 5.1.1 Langmuir Isotherm

1 $$q_{e}=\frac{q_{m}K_{L}C_{e}}{1+K_{L}C_{e}}$$

where *q*_m_ (mg. g^–1^) is the maximum monolayer adsorption capacity and *K*_L_ (L mg^–1^) is the Langmuir affinity constant. The model assumes monolayer adsorption on a homogeneous surface with energetically equivalent, noninteracting sites.^49^ Among the 1022 papers, the most frequently applied isotherm in clay-based PW studies is the red cluster in the VOSviewer network, which has dominated continuously from 1996 to 2025 (Figure 8). This bibliometric dominance mirrors findings from both the tetracycline adsorption bibliometric review (2015–2025, 567 papers)^48^ and the seaweed biosorption analysis (2014–2024, 369 papers),^50^ which independently identifies Langmuir as the canonical benchmark model across the adsorption literature. Its prevalence in this corpus, therefore, reflects field-wide methodological standardization rather than clay-specific surface behavior.^51^

#### 5.1.2 Freundlich Isotherm

2 $$q_{e}=K_{F}C_{e}\frac{1}{n}$$

where *K*_F_ indicates adsorption capacity and $\frac{1}{n}$ is the heterogeneity factor; a value of $\frac{1}{n}$ indicates favorable adsorption, while values approaching zero reflect a highly heterogeneous surface.^49^ The Freundlich model is the second-most-applied isotherm in this corpus, appearing predominantly in studies involving natural clays, bentonite, kaolinite, and montmorillonite, corresponding to the blue and green keyword clusters (Figure 8). Its rising frequency post-2016 (Figure 3) coincides with the SDG-driven surge in research exploring heterogeneous clay surfaces and modified clay-biosorbent hybrid materials. The seaweed biosorption bibliometric review^50^ reports the same Langmuir–Freundlich codominance across all categories of low-cost natural adsorbents, confirming both models as field-wide standards.^52^

#### 5.1.3 Sips Isotherm

The Sips isotherm combines Freundlich-type surface heterogeneity at low concentrations with Langmuir-type saturation at high concentrations, making it well suited to the complex, multicomponent chemistry of PW. Its usage in the corpus increased after 2018 (Figure 3), coinciding with growing research on composite clays and PW from hydraulic fracturing and oil-sands operations. This trend aligns with the tetracycline adsorption bibliometric review,^48^ which attributes the rise of hybrid isotherms to the recognized inadequacy of classical Langmuir and Freundlich models for describing real, multicomponent wastewater systems.^53^

The Dubinin–Radushkevich (DR) Isotherm

3 $$q_{e}=q_{s}e^{-K_{DR}\varepsilon^{2}}$$

4 $$\varepsilon =RT\;\ln\left(1+1C_{e}\right)$$

where *q*_s_ (mg g^–1^) is the theoretical saturation capacity, *K*_DR_ (mol^2^ kJ^–2^) is a constant related to mean free energy of adsorption, *R* is the gas constant (8.314 J mol^–1^ K^–1^), and *T* is the absolute temperature (K). The mean adsorption free energy is calculated as $E=1/\sqrt{2K_{DR}}$ ; values of *E* < 8 kJ mol^–1^ indicate physisorption governed by van der Waals forces, while *E* > 16 kJ mol^–1^ suggests chemisorption.^54^ Unlike purely empirical models, the DR isotherm provides mechanistic insight by distinguishing between physical and chemical adsorption, a distinction that is particularly relevant for modified clay composites. Within this corpus, the DR isotherm is increasingly reported in studies of surface-engineered clay composites, consistent with the green VOSviewer cluster (Figure 8), in which material modification shifts adsorption from weak van der Waals interactions to stronger ion-exchange and surface-complexation mechanisms. This bibliometric pattern confirms that DR model adoption tracks the field’s gradual transition from natural to functionalized clay systems.^54^

#### 5.1.4 Temkin Isotherm

5 $$q_{e}=\frac{RT}{b_{T}}\;\ln A_{T}C_{e}$$

where *A*_T_ (L g^–1^) is the equilibrium binding constant, *b*_T_ (J mol^–1^) is the Temkin constant related to the heat of adsorption, *R* is the gas constant, and *T* is the absolute temperature. The *A*_T_ and *b*_T_ constants are obtained from a linear plot of *q*_e_ versus ln (*C*_e_). The Temkin model assumes that the heat of adsorption decreases linearly with increasing surface coverage due to adsorbate–adsorbate interactions, making it suitable for systems with indirect surface interactions, which are increasingly encountered in high-concentration, multipollutant PW matrices.^49,55^ In the corpus, the Temkin isotherm remains the least frequently applied of the five models; however, its occurrence increases in post-2019 studies involving high-salinity PW and multimetal systems (Figure 3), reflecting a growing recognition of adsorbate interaction effects under realistic field conditions.

### 5.2 Kinetics

Kinetic modeling is essential for quantifying contaminant removal rates during PW treatment and for elucidating the underlying transport mechanisms. It provides critical information for system design, performance prediction, and process scale-up. Adsorption kinetics are typically determined through time-dependent batch experiments in which residual solute concentrations are measured at defined intervals, and the resulting data are fitted to mechanistic models to identify whether the rate-limiting step is governed by surface reactions, film diffusion, or intraparticle diffusion.^53,54^ In the 1022 papers, kinetic modeling forms an integral component of the red VOSviewer cluster (Figure 8), appearing consistently alongside isotherm analysis across all major temporal phases of the literature.

#### 5.2.1 Pseudo-First-Order (PFO) Model

6 $$\ln\left(q_{e}-q_{t}\right)=\ln\;q_{e}-k_{1}t$$

where *q*_e_ (mg g^–1^) and *q*_*t*_ (mg g^–1^) are the adsorption capacities at equilibrium and time *t*, respectively, and *k*_1_ (min^–1^) is the rate constant. The PFO model assumes that the adsorption rate is directly proportional to the number of vacant adsorption sites, making it particularly suitable for describing physisorption processes dominated by weak van der Waals forces.^56^ While the PFO model often provides a good fit for the initial, rapid phase of adsorption, it typically underestimates adsorption behavior at later stages as equilibrium is approached.^51^ Bibliometrically, PFO appears less frequently as the best-fit model in this corpus compared to PSO, consistent with the dominance of chemisorptive clay composites across the post-2016 literature.^51^

#### 5.2.2 Pseudo-Second-Order (PSO)

7 $$\frac{t}{q_{t}}=\frac{1}{k_{2}q_{e}^{2}}+\frac{t}{q_{e}}$$

where *k*_2_ (g mg^–1^ min^–1^) is the PSO rate constant. The model assumes chemisorption involving valence forces or electron sharing between the adsorbent and the adsorbate.^57^ The PSO model is the most frequently reported best-fit kinetic model in this corpus across the entire 1996–2025 period, a prevalence that mirrors findings in the tetracycline adsorption bibliometric review,^48^ where PSO dominates 567 studies. However, recent critiques corroborated by the seaweed biosorption bibliometric analysis^50^ suggest that this statistical superiority may partly reflect an inherent mathematical fitting advantage of the PSO linearization over other models, rather than universally confirming chemisorption as the underlying mechanism.^49^ This caveat is now increasingly acknowledged in the post-2020 literature within this corpus.

#### 5.2.3 Elovich Model

The Elovich model is widely applied to adsorption systems with highly heterogeneous surfaces, where the process involves variations in the activation energy. It is expressed as

8 $$q_{t}=\frac{1}{\beta }\;\ln\left(\alpha \beta \right)+\frac{1}{\beta }\;\ln t$$

where α (mg g^–1^ min^–1^) is the initial adsorption rate and β (g mg^–1^) is associated with surface coverage and activation energy.^51^ The Elovich model is applied to heterogeneous surfaces, where adsorption involves a distribution of activation energies, with rates declining progressively as surface coverage increases. In the corpus, Elovich model adoption increases in studies involving engineered clay composites and surface-modified bentonite, consistent with the transition from homogeneous natural clays to heterogeneous composite materials reflected in the expanding green VOSviewer cluster (Figure 8). This bibliometric pattern confirms that Elovich model usage tracks the field’s material complexity, as also reported in the seaweed biosorption review for composite biosorbent systems.^50^

#### 5.2.4 Intraparticle Diffusion Model (Weber–Morris)

9 $$q_{t}=k_{p}t^{0.5}+C$$

where *k*_p_ (mg g^–1^ min^–0.5^) is the intraparticle diffusion rate constant, and *C* (mg g^–1^) reflects the boundary layer thickness. If the plot of *q*_*t*_ versus *t* ^0.5^ is linear and passes through the origin, then intraparticle diffusion is considered the sole rate-limiting step. However, multilinear plots common in clay composite studies within this corpus indicate multiple sequential stages: external film diffusion, intraparticle pore diffusion, and final equilibrium, highlighting the structural complexity of porous composite clay adsorbents.^56^ The frequency of IPD model application in this corpus rises notably post-2016, coinciding with the proliferation of hierarchically porous clay composites in the literature, a parallel trend reported in the seaweed biosorption bibliometric review for analogous porous biosorbent structures.^50^

## 6 Contribution to SDGs

The use of clay-based materials in PW treatment supports climate action, promotes sustainable resource use, improves water quality, and protects ecosystems, all of which align with several SDGs. The radar chart in Figure 12 shows the distribution of clay-based PW treatment publications across all 17 SDGs, revealing an informative pattern. SDG 6 (Clean Water and Sanitation) dominates with nearly 500 publications, far exceeding other goals and indicating a primary focus on water quality and sanitation. This concentration matches the subject matter, but the disparity is notable: the gap between SDG 6 and the next most-represented goals, SDG 9 (Industry, Innovation, and Infrastructure), SDG 12 (Responsible Consumption and Production), SDG 13 (Climate Action), SDG 14 (Life Below Water), and SDG 15 (Life on Land), is large, with these goals showing only marginal mention near the center of the chart. Even more striking is the near absence of publications linking clay-based PW treatment to SDGs 1, 2, 3, 4, 5, 7, 8, 10, 11, and 16, which cover poverty, health, education, economic growth, and governance. This is not just a bibliometric observation; it also reflects a narrow framing within the research community regarding broader sustainability benefits. Although clay-based PW treatment has clear but underarticulated links to SDG 3 (health benefits from removing toxic contaminants in drinking water), SDG 8 (economic benefits of low-cost treatment for industrial operators in developing economies), and SDG 11 (sustainable urban water infrastructure), current SDG framing under-represents the broader societal value of this technology. Future studies should explicitly design research and reporting frameworks to measure contributions across a broader spectrum of SDGs, particularly in regions where PW contamination intersects with poverty, food security, and public health, thereby increasing both the scientific impact and the policy relevance. The following section provides a detailed description of the connections to the key SDGs.

**Figure 11:**
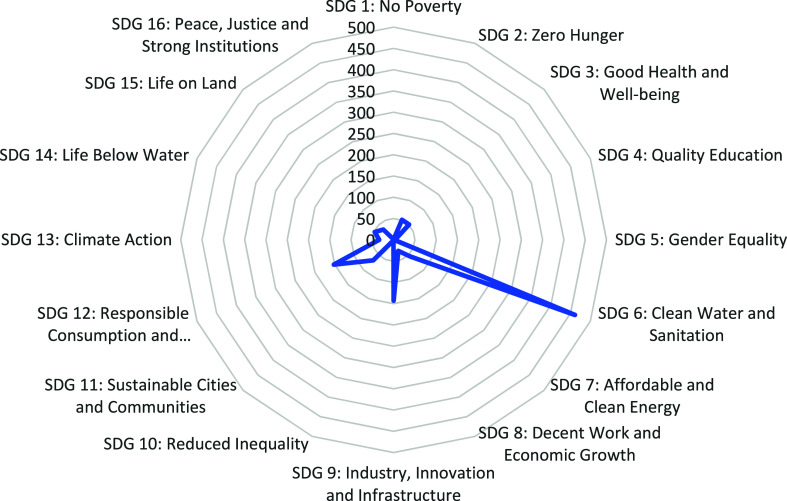
Top SDGs in the publications.

**Figure 12:**
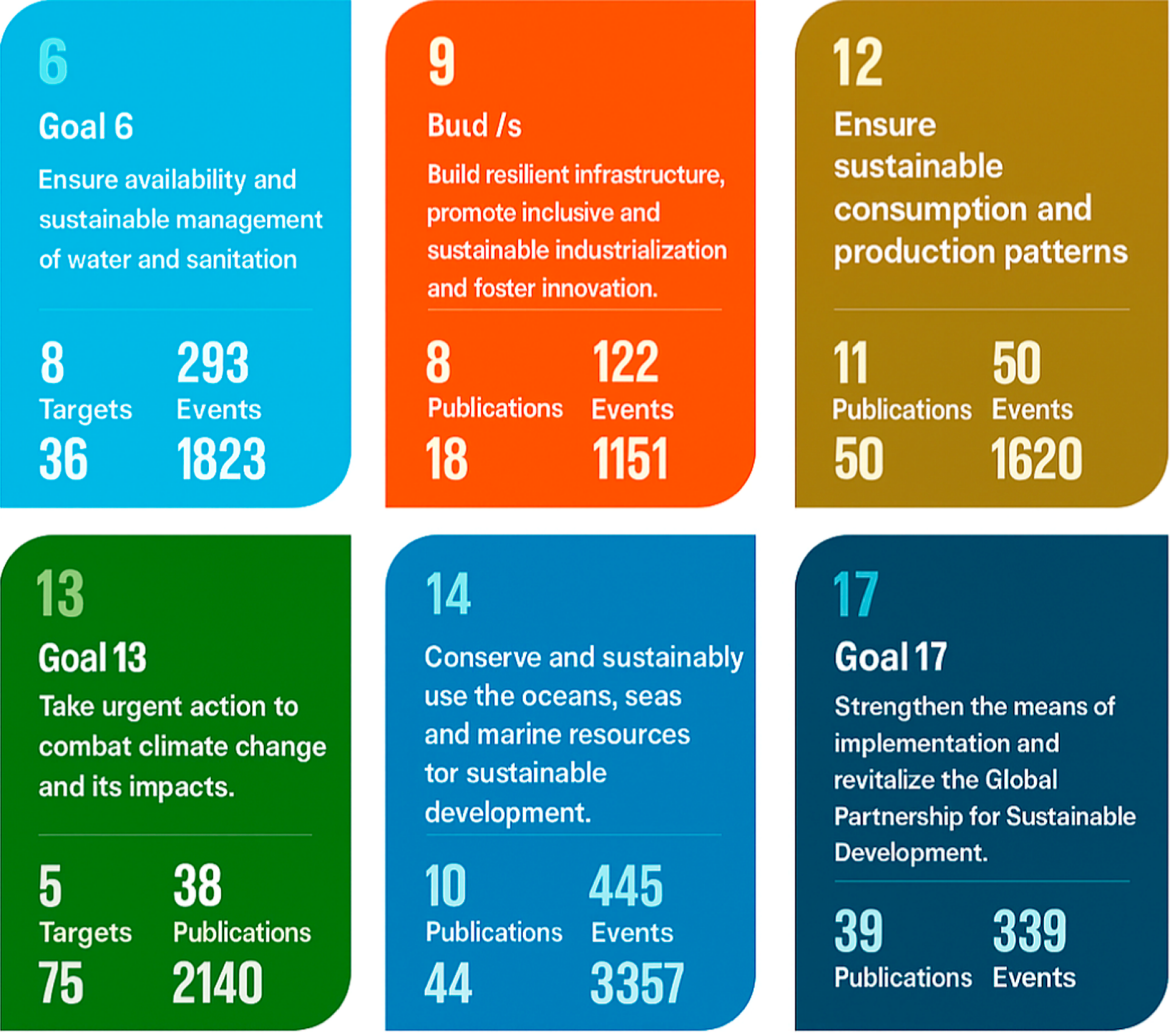
SDGs related to PW treatment using clay (Data adapted from the United Nations SDG database^5^).

### 6.1 SDG 6: Clean Water and Sanitation

Clay-based materials, especially natural clays such as bentonite and kaolin, are well-known for their ability to treat produced water (PW) by removing contaminants such as hydrocarbons, heavy metals, and organic pollutants. Data from the reviewed literature confirm this: bentonite and kaolin can remove heavy metals such as zinc at rates of up to 90%,^58^ supporting SDG Target 6.3 to reduce hazardous pollution and promote safe wastewater recycling by 2030. Their effectiveness in oil removal further emphasizes this connection. Natural clay achieved a crude oil sorption capacity of 6.9 g/g at pH 4 and 27 °C,^61^ and bentonite/iron oxide composites removed 71% of diesel oil from emulsions.^37^ Overall, 461 out of 1022 publications (45%) in the analyzed set align with SDG 6, confirming this goal as a key focus in the field.^30,59,60^

Beyond improving water quality, the ability to reuse clay adsorbents for at least three treatment cycles without a loss of efficacy directly supports SDG Target 6.4 by promoting cost-effective, repeated water recycling rather than single-use treatments. With clay’s widespread availability and lower cost than synthetic alternatives, clay-based treatment emerges as a feasible and scalable solution for industries striving to meet water sustainability objectives. Incorporating clay into PW treatment not only increases water safety but also promotes efficient resource utilization, significantly advancing SDG 6 and its related targets.^32,61–63^

### 6.2 SDG 9: Industry, Innovation, and Infrastructure

Clay-based materials serve as a sustainable and cost-effective alternative to synthetic agents in water treatment. In the bibliometric analysis, 143 publications (14% of the total) are explicitly linked to SDG 9 (see Figure 12), highlighting the field’s strong focus on industrial innovation and green infrastructure. Integrating these materials into treatment systems helps achieve SDG Target 9.4, which promotes upgrading infrastructure with resource-efficient, low-energy technologies. Using naturally abundant clays like bentonite and kaolin allows industries to modernize water treatment, conserving energy and lowering environmental impact compared to energy-intensive methods such as thermal distillation or reverse osmosis. This innovative aspect is also reflected in bibliometric analyses: the research cluster on clay’s mechanical and thermal properties, crucial for designing clay-based treatment systems, ranks in the 97.38th percentile globally in prominence. This indicates that clay-material engineering is among the most visible international research areas in this field. Such ongoing innovation supports SDG Target 9.5, encouraging the development of new eco-friendly industries and contributing to long-term environmental and economic sustainability.^63–65^

### 6.3 SDG 12: Responsible Consumption and Production

Clay, an abundant, naturally occurring material, offers a sustainable, low-cost alternative to synthetic chemicals in water treatment. It is linked to 152 publications (15% of the total), making it the second-most-represented goal in bibliometric analysis (Figure 11). This supports SDG Target 12.2, which emphasizes the sustainable management and efficient use of natural resources: unlike activated carbon or synthetic polymer adsorbents, which need energy-intensive manufacturing, natural clay minerals require minimal processing and are available worldwide at much lower costs. The ability to reuse clay adsorbents over at least three treatment cycles without losing efficiency further reduces material consumption per unit of treated water, directly supporting responsible production practices.^36^ Clay’s strong adsorption capacity for heavy metals (up to 90% removal for zinc^58^) and hydrocarbons (71% diesel oil removal^37^) helps achieve SDG Target 12.4 by minimizing hazardous releases into industrial effluents and promoting safer waste management. By aligning with these SDG targets, clay-based water treatment promotes sustainable resource management and responsible production in the water sector.^66–68^

### 6.4 SDG 13: Climate Action

Using natural materials like clay in water treatment contributes to climate action by offering a low-emission, energy-efficient alternative to synthetic treatment chemicals, though the bibliometric analysis reveals this alignment remains underarticulated: only 34 publications (3.3% of the corpus) are explicitly linked to SDG 13 (Figure 11), representing a significant gap given the field’s inherent environmental relevance. Clay-based treatment methods require minimal energy for production and application, in contrast to thermally activated adsorbents, such as activated carbon, whose production is highly energy-intensive. This reduces greenhouse gas emissions associated with water purification operations and aligns with SDG Target 13.2’s emphasis on integrating climate considerations into industrial practices. The growing adoption of these materials also supports SDG Target 13.3 by fostering climate-conscious approaches within the oil and gas sector. Critically, the low representation of SDG 13 in the current corpus indicates that researchers have not yet systematically quantified the carbon footprint or lifecycle emission reductions achieved through clay-based PW treatment, a gap that future studies should address through dedicated life cycle assessment (LCA) methodologies to strengthen this SDG linkage with concrete emission reduction data.^69–71^

### 6.5 SDG 14: Life Below Water and SDG 15: Life on Land

Clay-based treatment methods play a crucial role in protecting ecosystems by effectively removing harmful substances from PW before discharge into the environment. This supports SDG Target 14.1, which aims to reduce marine pollution caused by land-based activities. By preventing contaminants such as hydrocarbons and heavy metals from reaching oceans and seas, clay treatment helps safeguard aquatic life and maintain marine biodiversity.^72,73^ Despite this clear mechanistic alignment, only 48 publications (4.7%) are explicitly linked to SDG 14 (Figure 11), indicating that the marine ecosystem protection dimension of clay-based PW treatment is substantially under-reported in the literature. Likewise, SDG Target 15.1 is supported by clay treatment, which has demonstrated the capacity to intercept hydrocarbons and heavy metals before they enter soil systems, yet only 50 publications (4.9%) are linked to SDG 15, suggesting that outcomes for terrestrial ecosystem protection are equally underrepresented. Both of these gaps highlight a clear opportunity: future studies reporting contaminant removal performance should explicitly frame their findings in terms of marine and terrestrial ecosystem protection metrics to strengthen the SDG 14 and 15 evidence base.^74,75^

### 6.6 SDG 17: Partnerships for the Goals

Collaboration among governments, industries, and academic institutions is essential for increasing the adoption of clay-based solutions and fostering innovation in water treatment technologies. The bibliometric analysis offers clear evidence of this collaborative infrastructure. The institutional coauthorship network (Figure 5) highlights the Chinese Academy of Sciences, CSIC Spain, and the USDA Agricultural Research Service as the three most active hubs across Asia, Europe, and North America, exemplifying SDG Target 17.6, which encourages cross-regional technology sharing and capacity building for sustainable initiatives. China’s contribution of about 12.7% to total output, supported by national programs such as the NSFC, further demonstrates the government-academia partnership aligned with SDG Target 17.17, which promotes cross-sector collaboration to encourage eco-friendly innovations. Through such joint efforts, stakeholders have improved the understanding, development, and implementation of clay-based treatment methods, helping to integrate them into global markets and supporting the transition toward environmentally responsible industrial practices. Expanding this collaborative model to include research institutions in water-stressed developing nations where PW contamination poses the greatest risks presents the most direct way to ensure that technical improvements lead to tangible water security benefits.^5^

## 7 Future Directions

Future research on PW treatment using clay should focus on aligning more closely with the SDGs by developing frameworks to evaluate the environmental and socioeconomic impacts. Advances in material science, such as enhancing natural clays with nanomaterials or biobased additives, can boost adsorption and regeneration capabilities. Combining clay-based systems with hybrid technologies, such as advanced oxidation, membrane, and biological treatments, and supporting them with AI-driven optimization can significantly improve efficiency. Performing comprehensive life-cycle and techno-economic assessments is crucial to ensure sustainability and cost-effectiveness and to guide resource recovery and the safe disposal of spent clays. Additionally, addressing emerging contaminants, such as PFAS and microplastics, and evaluating system resilience to climate change will be vital for long-term effectiveness and global water security.

## 8 Conclusion

Clay-based treatment methods have attracted significant attention due to their high adsorption capacity, affordability, and environmental friendliness. Clays such as bentonite and montmorillonite can be used in their natural form or as modified composites to target specific contaminants. According to this bibliometric study, which analyzes keyword trends, research categories, and collaboration networks, there has been substantial global growth and development in research on clay-based PW treatment. By examining over 1022 publications using Scopus, WoS, and VOSviewer for data collection and network visualization, key research fields and influential contributors were identified. The analysis reveals that China is a global leader in clay-based PW treatment research, reflecting its significant investment and leadership in this field. Keyword mapping further indicates that adsorption remains the primary treatment method, with a strong focus on clay-based materials for water purification. To advance progress in this field, increased investment in R&D, infrastructure, and technology transfer is crucial, along with the development of sustainable business models that promote global adoption. These efforts will improve treatment efficiency and support circular water management and environmental sustainability.

## Data Availability

The bibliographic data analyzed in this study were retrieved from Scopus and WoS using the search queries and inclusion criteria reported in Section 3. The searches were executed, and the records were exported in April 2025; because Scopus and WoS are continuously updated, results may vary if the same query is run on a different date. The underlying bibliographic records are publicly accessible via Scopus and Web of Science (subject to user/institutional access). The processed/derived data generated during the analysis (e.g., publication counts and network matrices used for mapping) and the VOSviewer map files can be made available by the corresponding author upon reasonable request. VOSviewer is freely available software.
